# Optimizing Suitable Antibiotics for Bacterium Control in Micropropagation of Cherry Rootstock Using a Modified Leaf Disk Diffusion Method and E Test

**DOI:** 10.3390/plants8030066

**Published:** 2019-03-16

**Authors:** Chenglin Liang, Rendun Wu, Yu Han, Tian Wan, Yuliang Cai

**Affiliations:** College of Horticulture, Northwest A&F University, Yangling 712100, China; ljl2015lcl@163.com (C.L.); wrd002018@163.com (R.W.); 18592027262@163.com (Y.H.); wtian903@163.com (T.W.)

**Keywords:** antibiotics, bacterial contaminants, disk diffusion, E test, in vitro plant culture

## Abstract

Bacterial contamination is a major and constant threat to the establishment and subculture of in vitro plant culture. In this study, we used a slightly modified qualitative disk diffusion method to screen optimal antibiotics to control the growth of bacterial contaminants isolated from explants of cherry rootstock ‘Gisela 6’. Bacterial susceptibility to eight different antibiotics was tested. The results showed that tetracycline was the most effective antibiotic for controlling bacterial growth; cefotaxime, carbenicillin, kanamycin, and streptomycin were less effective, whereas ampicillin, penicillin, and cefazolin did not inhibit growth of the isolated bacteria. Using the quantitative E test, the minimal inhibitory concentration (MIC) of tetracycline was determined to be 1.0 µg mL^−1^. We also measured the F_v_/F_m_ values, chlorophyll content, and enzymatic activity of superoxide dismutase and peroxidase to explore the effect of different tetracycline concentrations, 0, 0.064, 0.5, 1.0, 16, and 256 µg mL^−1^, on the growth of bacteria and explants over 30 days. Results indicated that 1.0 µg mL^−1^ tetracycline was effective in restricting bacterial growth, with non-significant negative effects on explants at low concentrations, but were enhanced negative effects at high concentrations. The application of the disk diffusion method and E test enabled the identification of an antibiotic and its MIC value effective for eliminating bacterial contaminants while causing minimal damage to explants, indicating a high potential of these methods to control bacterial contaminants in in vitro plant culture.

## 1. Introduction

In vitro plant culture is an important tool in both basic and applied studies as well as in commercial settings [1], and has been used as the core technology for the mass propagation, conservation, and genetic manipulation of tissues in plant biology [2]. The ideal in vitro plant materials are bacterium-free. However, research and commercial in vitro culture laboratories are often beset by bacterial contamination [3]. Sources of in vitro bacterial contamination include infected explants, inadequate in vitro techniques, and poor laboratory environments [4,5,6]. Bacterial contaminants are either epiphytic or endophytic in nature [7]. For some valuable explant materials, it’s necessary to eliminate the bacterial contamination and reduce unnecessary losses. Epiphytic bacteria inhabit plant surfaces [8], and chemical disinfectants are generally used to eradicate these bacteria. By contrast, endophytic bacteria reside within plant tissues and are difficult to eliminate using simple surface sterilization methods [9]. Endophytic microbial contaminants in plant tissue culture often reduce the rate of tissue growth and multiplication, leading to tissue death in some cases [7].

Due to the adverse effects of endophytic bacteria on in vitro plant culture, preventing, reducing, or eliminating bacterial contamination is essential, which often requires the use of antibiotics [7,10,11]. Broad-spectrum antibiotics are used when the nature of the bacteria is unknown [12]. Long-term or repetitive antibiotic treatments can cause bacterial resistance. Additionally, long-term use of antibiotics at high concentrations in in vitro culture medium is generally not recommended because of potential toxicity and other negative side effects of antibiotics on the growth of explants [11]. Sometimes, it is very challenging to remove internal bacterial contaminants. Thus, effective and cheap antibiotics are needed. Previously, bacteria have been identified using conventional phenotyping [5,13] or genotyping by sequencing 16S ribosomal DNA for identifying many plant pathogens [7,14]. However, because of the innumerable species of bacterial pathogens, molecular characterization of these bacteria may not be sufficient for the optimal selection of antibiotics in a short time.

Currently, the disk diffusion method and E test are the most popular manual techniques for antibiotic susceptibility testing in many clinical microbiology laboratories because of their simplicity, reproducibility, and low cost [15]. The diffusion disk and E test were used in this study to develop an efficient and reliable method for screening antibiotics to better control contamination of in vitro plant culture without identification of bacterial strains. We also measured the ratio of the variable fluorescence (F_v_) to maximum fluorescence (F_m_) of cherry rootstock ‘Gisela 6’ (*Prunus cerasus* L. × *P. canescens* L.) explant leaves, chlorophyll content, and the activity of peroxidase (POD) and superoxide dismutase (SOD) to explore the effect of different antibiotic concentrations on the growth of explants over 30 days.

## 2. Materials and Methods

### 2.1. Plant Materials

In the spring of 2016, the shoots of cherry rootstock ‘Gisela 6’ were collected from a sweet cherry (*P. avium* L.) orchard in Zhouzhi, Xi’an, Shaanxi, China. The surface of shoots were rinsed with running water and then washed three times with sterile distilled water. The shoots were sterilized with 75% (*v*/*v*) ethyl alcohol for 40 s, and then rinsed three times with sterile distilled water. The shoots were then washed with 2% (*v*/*v*) sodium hypochlorite for 12 min and rinsed six times with sterile distilled water, and then dried on sterile filter paper. The shoots were cultured in solid Murashige and Skoog (MS) medium supplemented with 0.5 mg L^−1^ 6-benzylaminopurine (6-BA) and 0.1 mg L^−1^ indole-3-butyric acid (IBA). The MS medium contained 30 g L^−1^ sucrose and 7 g L^−1^ agar with pH 5.8. The explants were cultured in 240 mL glass tissue culture jars and were grown in a tissue culture room under 2000 lux light intensity, at 23 ± 2 °C, and 16 h light/8 h dark photoperiod. ‘Gisela 6’ explants were obtained by subculture.

### 2.2. Preparation of Bacterial Inoculum

When some ‘Gisela 6’ explants were contaminated, the bacteria easily spread to the medium. These bacteria were then isolated from the MS medium and were directly inoculated in lysogeny broth (LB) medium using an inoculation loop. These bacteria were then used for the disk diffusion method and E test. The sterile solid MS medium was inoculated with 100 μL of 1 × 10^8^ colony forming units (cfu) mL^−1^.

The uncontaminated explants with uniform growth were surface sterilized with 75% (*v*/*v*) ethyl alcohol for 40 s, washed in sterile distilled water three times, dried on sterile filter paper, and then placed on either sterile solid MS medium or inoculated solid MS medium. The explants placed on inoculated media (100 μL of 1 × 10^8^ colony forming units (cfu) mL^−1^) were labeled as the bacteria (B) group, and those placed on the same batch of solid MS media without bacterial inoculum were labeled as the control (CK) group.

### 2.3. Disk Diffusion Method

The antibiotic susceptibility test was performed using the disk diffusion method as described by Jorgensen and Ferraro (2009) [16], with slight modifications. For this test, 100 μL of bacterial inoculum (1 × 10^8^ cfu mL^−1^) was evenly spread on the surface of a 90 mm diameter LB-agar plate. When the surface of the plate dried, four commercial antimicrobial susceptibility disks (Hangzhou Microbial Reagent Co., Ltd., Hangzhou, China), each with a fixed antibiotic concentration, were placed on the inoculated agar surface. A total of eight antibiotics (kanamycin, streptomycin, ampicillin, cefotaxime, penicillin, cefazolin, tetracycline, and carbenicillin) were tested against this bacterial inoculum. Plates were incubated at 28 °C for 16–24 h. The inhibition zone surrounding each antibiotic disk was measured to the nearest millimeter.

### 2.4. E Test

The E test was performed as described by Luber et al. (2003) [17], with slight modifications. Commercial plastic E test strips (38 mm × 5 mm × 2 mm; Biokont, Wenzhou, China) with an exponential concentration gradient of dried and stabilized tetracycline, 256, 128, 64, 32, 16, 8, 4, 2, 1, 0.5, 0.25, 0.125, and 0.064 µg mL^−1^, were used. The minimal inhibitory concentration (MIC) value of tetracycline was read directly from the E test strip as the point at which a teardrop-shaped zone of inhibition intersected the MIC scale on the strip.

### 2.5. Antibiotic Treatment of ‘Gisela 6’ Explants

The explants in group B were cultured on inoculated MS medium for three days. Subsequently, explants were transferred to tissue culture jars containing solid MS medium supplemented with six different tetracycline concentrations, 0, 0.064, 0.5, 1.0, 16, and 256 µg mL^−1^, with four explants per jar. Depending on the antibiotic concentration of the media used to culture group B explants, they were designated as B-0, B-0.064, B-0.5, B-1.0, B-16, and B-256 and were treated for 0, 5, 10, 15, 20, 25, and 30 days. Group CK (control) explants were cultured on uninoculated media and were not subjected to antibiotic treatment.

### 2.6. Chlorophyll Fluorescence Imaging and F_v_/F_m_ Measurement

Plants were dark-adapted for at least 30 min before being transferred to the imager. To compensate for the brief light exposure during the transfer from the dark incubation container to the imager, plants were sealed inside the darkened fluorescence imager, Open FluorCam FC 800-O (PSI, Brno, Czech Republic), for 2 min before imaging [18]. Two explants in one tissue culture jar were placed inside the fluorescence imager and fluorescent images of individual apical leaves from explants in group CK (without antibiotic treatment) and group B (treated) were captured using the following settings: Light Sources: Flashes, Global Light Settings: Act1 100%, Super 20%, Act2 20%, El. Shutter (1): 20 μs, Sensitivity: 50%, Filter: A: ChlF. These images were analyzed using the Fluorcam7 software (version 1.5.0.46, Photon Systems Instruments, Brno, Czech Republic). The program first displayed a visual image, which was a black and white image of the leaf under actinic light with green false color added; this image was used to determine the time when visible symptoms developed. The chlorophyll fluorescence parameter, F_v_/F_m_ reflects the maximum quantum efficiency of photosystem II (PSII). Values of F_v_/F_m_ were quantified from the false-color images of parameters F_o_, F_m_, F_m_′ generated after 60 s of actinic light exposure. The minimum and maximum color ranges for the false color F_v_/F_m_ images were standardized to 0.1 and 0.8, respectively; using the Fluorcam7 software to ensure consistency in the false color scale between leaves. All false color images and the visual images were saved as BMP image data files and compressed into TAR archives in FluorCam7.

### 2.7. Measurement of Chlorophyll Content

To measure the relative chlorophyll content of tetracycline treated leaves, leaf tissues (0.1 g) treated with tetracycline for 0, 5, 10, 15, 20, 25, and 30 days were extracted with 80% (*v*/*v*) acetone. The homogenized mixture was centrifuged at 3000× *g* for 10 min. The optical density of the supernatant was measured at 645, 663, and 652 nm using a spectrophotometer (Type: 1510, Thermo Fisher Scientific Oy, Vantaa, Finland) to determine the content of chlorophyll-a, chlorophyll-b, and total chlorophyll, respectively [19]. Leaves of four plants were analyzed per treatment, and each treatment was replicated three times.

### 2.8. Enzyme Extraction and Activity Assays

Leaf samples (0.1 g) were ground in 1% (*w*/*v*) polyvinylpolypyrrolidone (PVP) using pre-chilled mortar and pestle, and homogenized in 1.2 mL of 50 mM potassium phosphate buffer (pH 7.8) containing 1 mM ethylenediaminetetraacetic acid (EDTA) and 0.3% Triton X-100 [20]. The enzymatic activity of POD (EC 1.11.1.7) was assayed at 470 nm in a reaction mixture (1.0 mL) containing 100 mM potassium phosphate buffer (pH 6.0), 16 mM guaiacol, 5 µL of 10% (*v*/*v*) hydrogen peroxide (H_2_O_2_), and the enzyme extract, as described previously [21]. The enzymatic activity of SOD (EC 1.15.1.1) was assayed under light conditions [22] in a reaction mixture (1 mL) containing 50 mM buffer, 65 mM methionine phosphate buffer, 0.5 mM nitrotetrazolium blue chloride, 0.1 mM riboflavin, 1 mM EDTA, and the enzyme solution. The activity of antioxidant enzymes was measured at 0, 5, 10, 15, 20, 25, and 30 days after tetracycline treatment. Leaves of four plants were analyzed per treatment, and each treatment was replicated three times.

### 2.9. Statistical Analysis

The data were analyzed via one-way ANOVA, followed by Duncan’s multiple range tests. A *p*-value < 0.05 indicated a significant difference, and data were presented as means ± standard deviation (SD) of three replicate samples, except for the chlorophyll fluorescence measurements, which involved four replicates.

## 3. Results

### 3.1. Determining the Antibiotic and Its Minimal Inhibitory Concentration (MIC) Effective in Controlling Bacterial Growth

**Figure 1:**
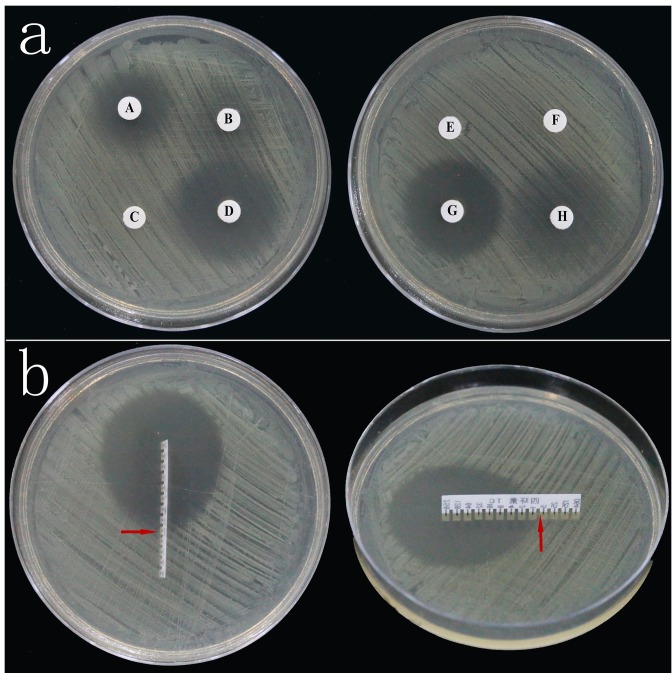
Disk diffusion method and E test. (**a**) Antibiotic susceptibility testing of bacteria using the disk diffusion method. The LB-agar plates were inoculated with bacterial culture, and four disks, each containing a different antibiotic, were incubated per plate. (A) Kanamycin, (B) Streptomycin, (C) Ampicillin, (D) Cefotaxime, (E) Penicillin, (F) Cefazolin, (G) Tetracycline, (H) Carbenicillin; (**b**) determination of the minimum inhibitory concentration (MIC) of tetracycline effective against bacterial contaminants using the E test. The E test strip with a series of tetracycline concentrations was placed on the LB-agar plate inoculated with bacterial culture. The MIC value of tetracycline was determined as the point at which the teardrop shaped zone of inhibition intersected the MIC scale inscribed on the strip (indicated with red arrows).

We used the disk diffusion method, a qualitative technique, to test the susceptibility of bacteria isolated from cherry rootstock ‘Gisela 6’ to eight antibiotics, including kanamycin, streptomycin, ampicillin, cefotaxime, penicillin, cefazolin, tetracycline, and carbenicillin. Results showed that tetracycline was most effective for inhibiting bacterial growth, followed by cefotaxime, carbenicillin, kanamycin, and streptomycin. Ampicillin, penicillin, and cefazolin did not inhibit bacterial growth (Figure 1a). Results of the E test showed that the MIC value of tetracycline was in the range of 0.5–1.0 µg mL^−1^ (Figure 1b). Because the tip of the inhibition zone pointed to a region between two different tetracycline concentrations, 0.5 and 1.0 µg mL^−1^, we added these to the concentration gradient for determining the optimal MIC value of tetracycline.

### 3.2. Effect of Tetracycline on the Growth of Bacteria and Explants

**Figure 2:**
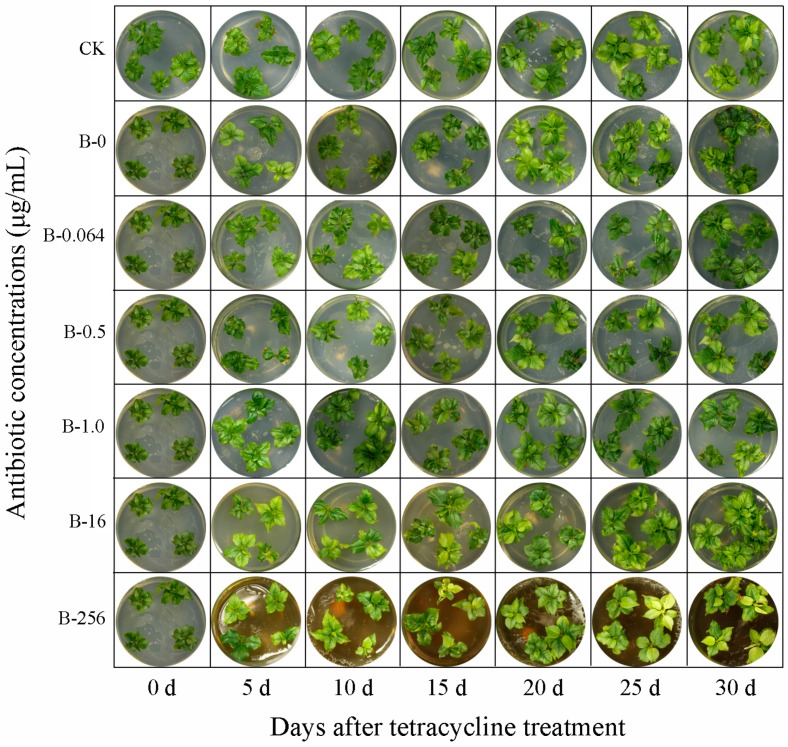
Evaluation of the effect of tetracycline on the growth of cherry rootstock ‘Gisela 6’ explants and bacterial inoculum. The explants were grown on solid Murashige and Skoog (MS) medium inoculated with a fixed concentration of bacterial inoculum and supplemented with variable concentrations of tetracycline: 0, 0.064, 0.5, 1.0, 16, and 256 μg mL^−1^, labeled as B-0, B-0.064, B-0.5, B-1.0, B-16, and B-256, respectively. Photographs were taken on 0, 5, 10, 15, 20, 25, and 30 days of tissue culture. Representative images are shown. Control group (CK), control explants without bacterial and tetracycline treatment.

**Figure 3:**
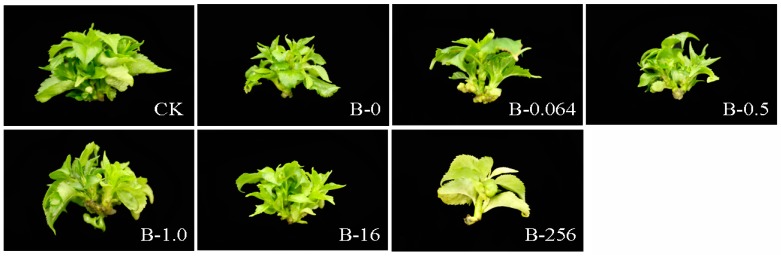
Images showing the differentiation of ‘Gisela 6’ explants treated with different concentrations of tetracycline: 0, 0.064, 0.5, 1.0, 16, and 256 μg mL^−1^, labeled as B-0, B-0.064, B-0.5, B-1.0, B-16, and B-256, respectively, on the 25th day of tissue culture. Representative images are shown. CK, control explants without bacterial and tetracycline treatment.

From the results of the E test strip, we monitored the growth of bacteria on explants treated with a wide range of tetracycline concentrations, 0, 0.064, 0.5, 1.0, 16, and 256 µg mL^−1^, for 0, 5, 10, 15, 20, 25, and 30 days. Results showed that tetracycline concentrations ≤0.5 µg mL^−1^ were ineffective, whereas concentrations ≥1.0 µg mL^−1^ were highly effective in eliminating bacteria (Figure 2). Notably, the leaves of explants turned yellow when treated with high tetracycline concentration (≥16 µg mL^−1^) for an extended period of time (≥5 d) (Figure 2). The group B explants showed no differentiation at the highest tetracycline concentration (256 µg mL^−1^) on the 25th day, whereas the differentiation of group B explants was slightly weaker than that of group CK explants at all other concentrations of tetracycline (Figure 3).

### 3.3. Chlorophyll Fluorescence Imaging and Quantification of F_v_/F_m_ Values

**Figure 4:**
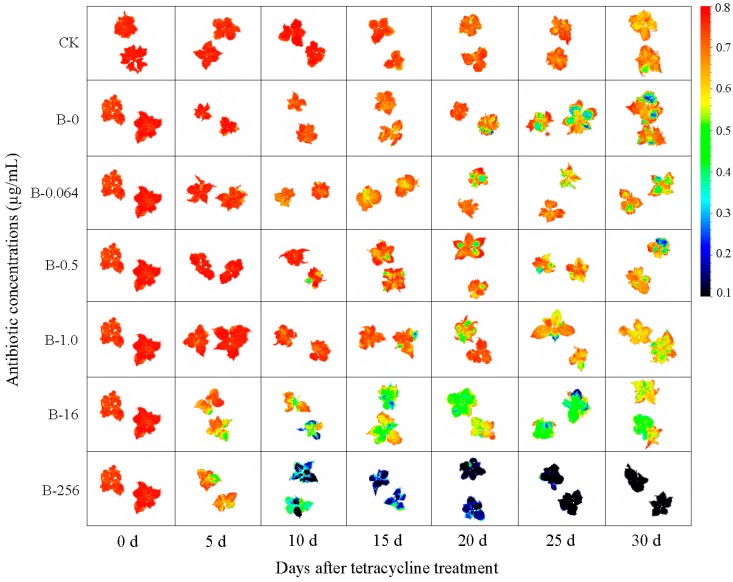
Chlorophyll fluorescence (F_v_/F_m_) images of ‘Gisela 6’ explants treated with different concentrations of tetracycline and grown for up to 30 days. Images of fluorescence parameters are displayed with the help of a false color code ranging from 0.1 (black) to 0.8 (red). Representative measurements are shown. B-0, B-0.064, B-0.5, B-1.0, B-16, and B-256 indicate 0, 0.064, 0.5, 1.0, 16, and 256 μg mL^−1^ tetracycline, respectively. CK, control explants without bacterial and tetracycline treatment.

To investigate the influence of antibiotic treatment on photosynthesis, changes in the photosynthetic activity of ‘Gisela 6’ explants were assessed using F_v_/F_m_ imaging after treatment with different concentrations of tetracycline. The minimum and maximum color ranges for false color F_v_/F_m_ images were standardized to 0.1 and 0.8, respectively, using the Fluorcam7 software to ensure consistency in the false color scale between leaves. The leaf color of explants in the CK group changed from red to orange after 15 days, whereas the leaves of explants in B-0, B-0.064, B-0.5, and B-1.0 groups showed this color change after 10 days (Figure 4). Leaves of explants in groups B-0 and B-0.064 appeared green on the 20th day, whereas those in groups B-0.5 and B-1.0 appeared green after 15 days. However, leaves of explants in groups B-0, B-0.064, B-0.5, and B-1.0 did not turn green over their entire surface until the 30th day. By contrast, leaves of explants in groups B-16 and B-256 appeared green on the 5th day; most of the leaves in B-16 group started turning green on the 15th day, and leaves in B-256 group started turning black on the 10th day (Figure 4).

**Figure 5:**
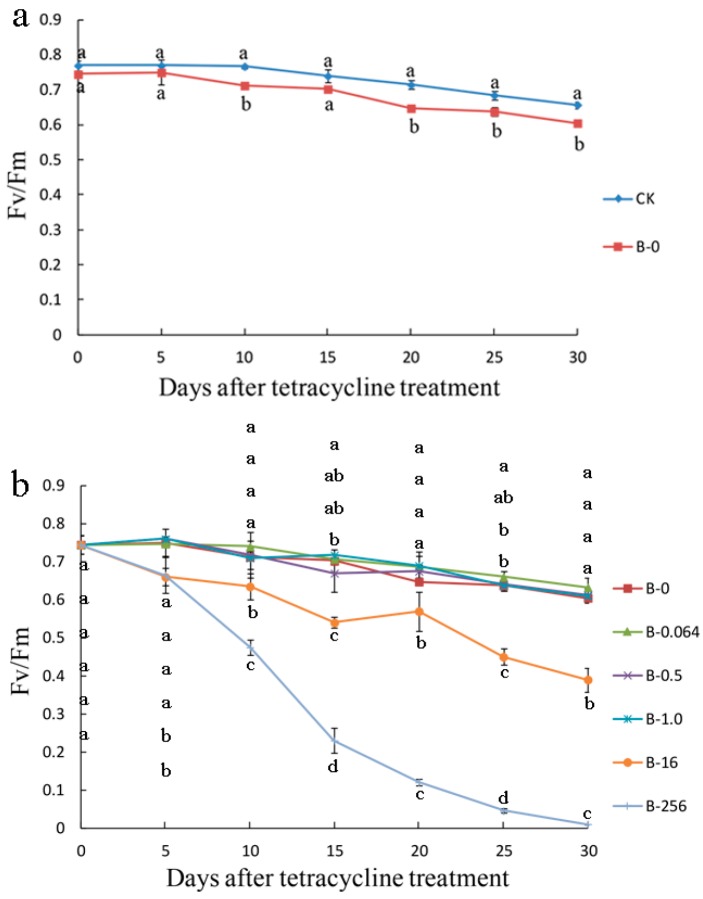
Maximum quantum efficiency (F_v_/F_m_) of photosystem II (PSII) of ‘Gisela 6’ explants. (**a**) F_v_/F_m_ of ‘Gisela 6’ explants treated with bacteria (B-0) for 30 days or without bacterial treatment (CK, control); (**b**) F_v_/F_m_ of ‘Gisela 6’ explants treated with different concentrations of tetracycline: 0, 0.064, 0.5, 1.0, 16, and 256 μg mL^−1^, labeled as B-0, B-0.064, B-0.5, B-1.0, B-16, and B-256, respectively for 30 days. Data represent means ± standard deviation (SD) of four replicate samples. Different letters indicate significant differences according to Duncan’s multiple range test (*p* < 0.05).

The F_v_/F_m_ values of B-0 leaves were lower than those of the CK leaves and showed a slight decline in both groups (Figure 5a). With the increase in the time of tetracycline treatment, the F_v_/F_m_ values of low concentration groups (B-0, B-0.064, B-0.5, and B-1.0) displayed the same trend and were >0.6 throughout the 30 days (Figure 5b). A marked decline was observed in the F_v_/F_m_ values of leaves of B-16 and B-256 groups; F_v_/F_m_ values of B-16 leaves decreased to 0.39 on the 30th day and those of B-256 leaves decreased to approximately zero on the 25th day (Figure 5b).

### 3.4. Effect of Tetracycline on Chlorophyll Content and Enzyme Activity

**Figure 6:**
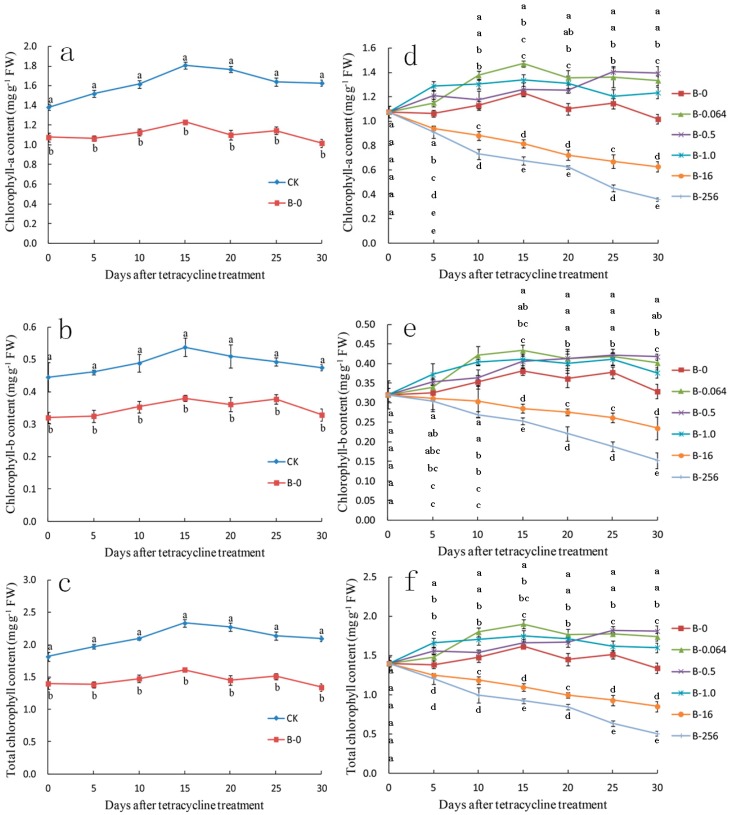
Changes in the chlorophyll content of ‘Gisela 6’ explants treated with (**a**–**c**) bacterial inoculum and (**d**–**f**) tetracycline for 30 days. (**a**,**d**) chlorophyll-a content; (**b**,**e**) chlorophyll-b content; (**c**,**f**) total chlorophyll content. CK, control explants without bacterial and tetracycline treatment; B, explants treated with bacterial inoculum; B-0, B-0.064, B-0.5, B-1.0, B-16, and B-256 represent explants treated with 0, 0.064, 0.5, 1.0, 16, and 256 μg mL^−1^ of tetracycline, respectively. Data represent means ± SD of three replicate samples. Different letters indicate significant differences according to Duncan’s multiple range test (*p* < 0.05).

To evaluate the effect of tetracycline on chlorophyll, we measured the chlorophyll content of leaves after 0, 5, 10, 15, 20, 25, and 30 days of tetracycline treatment (Figure 6). The content of chlorophyll-a, chlorophyll-b, and total chlorophyll showed the same trend. The chlorophyll content of the CK leaves was slightly higher than that of B-0 leaves (Figure 6a–c) and similar to that of B-0.064, B-0.5, and B-1.0 leaves. Compared with B-0 leaves, the chlorophyll content of B-0.064, B-0.5, and B-1.0 leaves was slightly high and that of B-16 and B-256 leaves was relatively low; among all B group leaves, the chlorophyll content of B-256 leaves was the lowest (Figure 6d–f).

**Figure 7:**
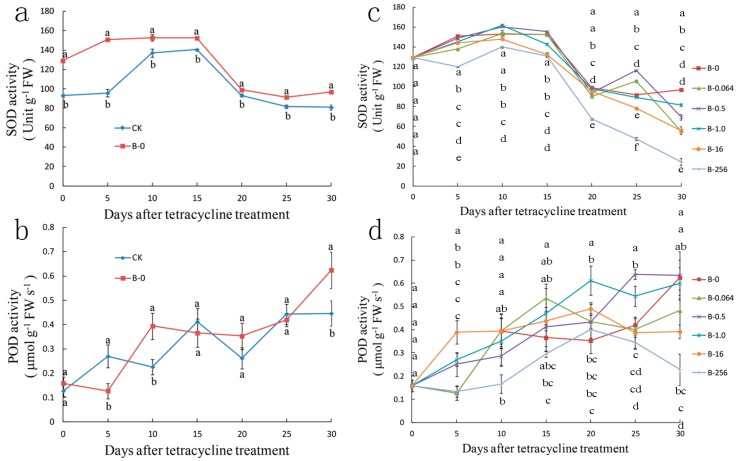
Effect of bacterial and tetracycline treatment of ‘Gisela 6’ explants on the enzymatic activity of (**a**,**c**) superoxide dismutase (SOD) and (**b**,**d**) peroxidase (POD) for 30 days. (**a**,**b**) enzymatic activity in ‘Gisela 6’ explants after bacterial treatment. (**c**,**d**) enzymatic activity in ‘Gisela 6’ explants treated with different concentrations of tetracycline. CK, control explants without bacterial and tetracycline treatment; B, explants treated with bacterial inoculum; B-0, B-0.064, B-0.5, B-1.0, B-16, and B-256 represent explants treated with 0, 0.064, 0.5, 1.0, 16, and 256 μg mL^−1^ of tetracycline, respectively. Data represent means ± SD of three replicate samples. Different letters indicate significant differences according to Duncan’s multiple range test (*p* < 0.05).

We also evaluated the effect of bacteria and tetracycline on the enzymatic activities of the antioxidant enzymes, POD and SOD (Figure 7). Compared with the CK explants, the SOD activity of B-0 explants was slightly higher (Figure 7a); however, no significant difference was observed between the CK and B-0 explants in POD activity (Figure 7b). The activity of SOD in the explants of group B showed a gradual initial increase followed by a decline; the SOD activity was the lowest in B-256 explants (Figure 7c). The POD activity of group B explants displayed a fluctuating ascending tendency (Figure 7d).

## 4. Discussion

Microbial contamination is one of the biggest problems in in vitro plant culture that directly affects the cost of production and preservation of valuable explants. Bacterial contaminants are particularly problematic, as they often appear only after several subcultures of seemingly clean cultures [7]. In this study, explants of the B-0 group were cultured on MS medium inoculated with bacteria for three days, and then transferred to sterile tissue culture jars. Results showed that the bacteria affected plant differentiation, the chlorophyll fluorescence parameter, chlorophyll content and SOD activity. To solve the problem of bacterial contamination, bacteria have been isolated from a diverse group of plants grown in vitro [23,24] and identified using molecular techniques to determine the most effective antibiotics [25,26]. In the study of Khan et al. (2018) [27], eight distinct bacterial isolates from in vitro plantlets of *Ficus indica* were identified by 16S rRNA sequence analysis, and nine different antibiotics were tested for their activity against the identified endophytes. These methods of bacterial isolation and identification are costly and time-consuming, but do not elucidate effective treatments to eliminate bacterial contamination of explants. Broad-spectrum antibiotics continue to be misused [12]. Therefore, in this study, we chose eight antibiotics to test in the disk diffusion test. This method is currently the most popular method used in many laboratories, and results of antibiotic susceptibility tested using this method are highly reproducible. The disk diffusion method is a simple test that does not require any special equipment; its results are qualitative in nature and are easy to interpret. The diameter of the inhibition zone surrounding the antibiotic disk is related to the susceptibility of the isolate and the rate of antibiotic diffusion through the agar medium. In this study, results of the disk diffusion method showed that tetracycline was most effective in controlling bacterial growth, followed by cefotaxime, carbenicillin, kanamycin, and streptomycin. Although the inhibition zones of cefotaxime and carbenicillin were similar in size to that of the tetracycline, bacteria were found growing in the inhibition zones of cefotaxime and carbenicillin, indicating that bacterial contaminants from ‘Gisela 6’ explants were mixed strains. Therefore, cefotaxime and carbenicillin were unable to completely control the bacterial contamination of ‘Gisela 6’ explants.

Since the disk diffusion method is a qualitative technique, we used the E test to determine the MIC value of tetracycline, which is a quantitative diffusion method and is faster than the disk diffusion method in producing results [17]. Results showed that the MIC value of tetracycline was in the range of 0.5–1.0 µg mL^−1^. Based on the effect of tetracycline on the growth of bacteria and explants, we determined 1.0 µg mL^−1^ as the optimal MIC value of tetracycline. This suggests that when the tip of the inhibition zone points toward a region between two different antibiotic concentrations, the larger value should be considered as the optimal MIC value. At high tetracycline concentration (≥16 µg mL^−1^), the MS medium darkened in color, and ‘Gisela 6’ explants grew weaker with yellow leaves. In plant in vitro culture, an important index of plant propagation ability is explant differentiation. Explant propagation was negatively affected at extremely high concentrations of tetracycline (256 µg mL^−1^) on the 25th day, whereas the effects on group B explants at all other concentrations of tetracycline were slightly weaker than that on group CK explants. Previous studies have shown that different antibiotics have variable effects on different plant tissues in vitro, and negative effects of antibiotics on explants are enhanced at higher concentrations [28,29]. These data are consistent with the results of our research.

The chlorophyll fluorescence parameter, F_v_/F_m_, reflects the maximum quantum efficiency of photosystem II (PSII) and has been widely used for early stress detection in plants [30,31]. Some studies showed that antibiotics were detrimental to the photosynthetic rate and chlorophyll biosynthesis of plants, which varied between different antibiotics and concentrations of antibiotics [32,33]. In this study, no significant differences were observed in the F_v_/F_m_ values of B-0, B-0.064, B-0.5 and B-1.0 groups at low tetracycline concentrations, whereas a marked decline was detected in the F_v_/F_m_ values of B-16 and B-256 groups. The maximum quantum efficiency of B-16 and B-256 groups declined rapidly on the 5th day, indicating that explants were damaged by high tetracycline concentrations. Adverse growing conditions influence chlorophyll metabolism and directly affect plant growth and yield [34]. One of the early responses to environmental challenges is an increase in the chlorophyll content [35]. This is consistent with our data showing a gradual initial increase in the chlorophyll content of explants at low tetracycline concentrations and a steady decline in chlorophyll content at high tetracycline concentrations.

When net photosynthesis decreases, excess excitation energy leads to impaired PSII function, an accumulation of reactive oxygen species, and oxidative damage [36]. Oxidative damage can be avoided or minimized through the function of antioxidant enzymes, such as SOD and POD. Our results showed that the SOD activity gradually increased initially and then declined, whereas the POD activity displayed a fluctuating ascending tendency.

## 5. Conclusions

This study used the disk diffusion method and E test to screen optimal antibiotics to control the growth of bacterial contaminants in plant in vitro culture. These methods enabled the identification of an antibiotic and its MIC value effective for eliminating bacterial contaminants while causing minimum damage to the explants. It is noteworthy that for the bacterial contaminants isolated from the explants of cherry rootstock ‘Gisela 6’ in this study, 1.0 µg mL^−1^ tetracycline was effective in restricting bacterial growth, with minimal negative effects on explants. Different explants may contain different bacterial contaminants, and even the same explant may develop different bacterial contamination, especially if acquired from the environment. This means that antibiotics required to control bacterial infection in other explant experiments may not be the same as those selected in this study. However, researchers can use the same protocols applied in this study to find the best antibiotic and concentration to control bacterial contamination in their in vitro plant culture.

## Abbreviations

MIC	Minimal inhibitory concentration
POD	Peroxidase
SOD	Superoxide dismutase
MS	Murashige and Skoog
6-BA	6-benzylaminopurine
IBA	Indole-3-butyric acid
LB	Lysogeny broth
CFU	Colony forming units
PVP	Polyvinylpolypyrrolidone
EDTA-Na_2_	Ethylenediaminetetraacetic acid disodium salt
CK	Control explants without bacterial and tetracycline treatment
B-0, B-0.064, B-0.5, B-1.0, B-16, B-256	0, 0.064, 0.5, 1.0, 16, and 256 μg mL^−1^ tetracycline
